# Human IFIT1 Inhibits mRNA Translation of Rubulaviruses but Not Other Members of the Paramyxoviridae Family

**DOI:** 10.1128/JVI.01056-16

**Published:** 2016-09-29

**Authors:** D. F. Young, J. Andrejeva, X. Li, F. Inesta-Vaquera, C. Dong, V. H. Cowling, S. Goodbourn, R. E. Randall

**Affiliations:** a School of Biology, Centre for Biomolecular Sciences, University of St. Andrews, St. Andrews, Fife, United Kingdom; b Norwich Medical School, University of East Anglia, Norwich Research Park, Norwich, United Kingdom; c School of Life Sciences, Centre for Gene Regulation and Expression, University of Dundee, Dundee, United Kingdom; d Institute for Infection and Immunity, St. George's, University of London, London, United Kingdom; Wake Forest University

## Abstract

We have previously shown that IFIT1 is primarily responsible for the antiviral action of interferon (IFN) alpha/beta against parainfluenza virus type 5 (PIV5), selectively inhibiting the translation of PIV5 mRNAs. Here we report that while PIV2, PIV5, and mumps virus (MuV) are sensitive to IFIT1, nonrubulavirus members of the paramyxoviridae such as PIV3, Sendai virus (SeV), and canine distemper virus (CDV) are resistant. The IFIT1 sensitivity of PIV5 was not rescued by coinfection with an IFIT1-resistant virus (PIV3), demonstrating that PIV3 does not specifically inhibit the antiviral activity of IFIT1 and that the inhibition of PIV5 mRNAs is regulated by *cis*-acting elements. We developed an *in vitro* translation system using purified human IFIT1 to further investigate the mechanism of action of IFIT1. While the translations of PIV2, PIV5, and MuV mRNAs were directly inhibited by IFIT1, the translations of PIV3, SeV, and CDV mRNAs were not. Using purified human mRNA-capping enzymes, we show biochemically that efficient inhibition by IFIT1 is dependent upon a 5′ guanosine nucleoside cap (which need not be N7 methylated) and that this sensitivity is partly abrogated by 2′O methylation of the cap 1 ribose. Intriguingly, PIV5 M mRNA, in contrast to NP mRNA, remained sensitive to inhibition by IFIT1 following *in vitro* 2′O methylation, suggesting that other structural features of mRNAs may influence their sensitivity to IFIT1. Thus, surprisingly, the viral polymerases (which have 2′-O-methyltransferase activity) of rubulaviruses do not protect these viruses from inhibition by IFIT1. Possible biological consequences of this are discussed.

**IMPORTANCE** Paramyxoviruses cause a wide variety of diseases, and yet most of their genes encode structural proteins and proteins involved in their replication cycle. Thus, the amount of genetic information that determines the type of disease that paramyxoviruses cause is relatively small. One factor that will influence disease outcomes is how they interact with innate host cell defenses, including the interferon (IFN) system. Here we show that different paramyxoviruses interact in distinct ways with cells in a preexisting IFN-induced antiviral state. Strikingly, all the rubulaviruses tested were sensitive to the antiviral action of ISG56/IFIT1, while all the other paramyxoviruses tested were resistant. We developed novel *in vitro* biochemical assays to investigate the mechanism of action of IFIT1, demonstrating that the mRNAs of rubulaviruses can be directly inhibited by IFIT1 and that this is at least partially because their mRNAs are not correctly methylated.

## INTRODUCTION

Paramyxoviruses are a large group of negative-sense single-stranded RNA viruses that cause a wide variety of animal and human diseases. The Paramyxoviridae family is divided into two subfamilies, the Paramyxovirinae and the Pneumovirinae subfamilies. The Paramyxovirinae are further subdivided into a number of genera, including Morbillivirus (e.g., Measles virus [MeV] and Canine distemper virus [CDV]), Respirovirus (e.g., Sendai virus [SeV] and Parainfluenza virus type 3 [PIV3]), and Rubulavirus (e.g., Mumps virus [MuV], PIV2, and PIV5). Paramyxoviruses are enveloped viruses; the viral glycoproteins protrude from the outer surface of the envelope and function to attach the viruses to their target cells. On the inner surface of the envelope is the matrix (M) protein, which is required for the structural integrity of the virion. The envelope surrounds a helical nucleocapsid, in which the nucleocapsid protein (NP) encapsidates genomic or antigenomic RNA. Associated with the nucleocapsid is the virally encoded polymerase complex. The viral polymerase both transcribes and replicates the viral genome. Viral mRNAs are capped and polyadenylated by the viral polymerase (for reviews of the molecular biology of paramyxoviruses, see references 1 and 2).

Despite their limited genetic information, the majority of paramyxoviruses encode small multifunctional accessory proteins that function to aid virus multiplication and block cellular antiviral defense mechanisms; typically, these proteins can block both the production of, and the signaling response to, interferons (IFNs) (for reviews, see references 3, 4, 5, 6, and 7). Significantly, the mechanisms of action of these multifunctional IFN antagonists differ from one virus to another. Undoubtedly, these properties and in general the manner in which paramyxoviruses interact with the IFN system and other innate defense mechanisms are likely to be major factors in determining the type of disease that each virus causes (8).

The IFN response is an extremely powerful antiviral defense system that, unless counteracted by viruses, will limit their replication to such a degree that they will not cause disease or be efficiently transmitted between susceptible hosts (8, 9). Infected cells detect the presence of viruses due to the production by viruses of molecules with molecular signatures (pathogen-associated molecular patterns [PAMPs]) such as double-stranded RNA (dsRNA), which activate the IFN induction cascade and result in the secretion of IFN-α/β from infected cells (9, 10). The release of IFN induces an antiviral state in neighboring uninfected cells by upregulating the expression of hundreds of interferon-stimulated genes (ISGs), many of which have direct or indirect antiviral activity (11). Most paramyxoviruses counteract the IFN responses by producing proteins that block IFN induction and/or IFN signaling by a variety of mechanisms (3–7). Furthermore, they tightly control viral transcription and replication, thereby limiting the production of PAMPs that may activate the IFN response (12, 13). Indeed, it is probably mistakes that viruses make during transcription and replication, such as the production of copy-back-defective interfering particles, that activate the IFN response (14–16; reviewed in reference 17). Nevertheless, the ability of paramyxoviruses to block the IFN response both in tissue culture cells and *in vivo* is not absolute, and some IFN-α/β will be produced (18, 19). Furthermore, IFN-γ, which can also induce an antiviral state in cells, will also be produced by activated subsets of lymphocytes (20). Therefore, it is inevitable that viruses will infect cells in a preexisting IFN-induced antiviral state, potentially limiting the speed of virus replication and spread. Although IFNs induce hundreds of ISGs, several ISGs with direct antiviral activity have been shown to be specific for families or groups of related viruses (11, 21, 22). With regard to the Paramyxoviridae family, we have previously shown that ISG56/IFIT1 (here referred to as IFIT1), which selectively inhibits translation, is the primary effector of the IFN-induced antiviral state that limits the replication of the rubulavirus PIV5 (23). Pretreatment of cells with IFN-α/β inhibits PIV5 protein synthesis but not cellular protein synthesis. This is because IFIT1 selectively inhibits the translation of PIV5 mRNAs but does not affect cellular mRNAs (23).

Mammalian mRNAs have an N-7 methyl guanosine (m^7^GpppN), termed cap 0, at their 5′ end that recruits factors involved in RNA processing and translation initiation. The first and second nucleosides of mammalian mRNAs are also methylated on the 2′ hydroxyl group of the ribose ring, generating cap 1 and cap 2, respectively. While cap 1 and cap 2 are not required for efficient mRNA translation, IFIT1 can inhibit the translation of mRNAs that lack cap 1 (24–27). IFIT1 also binds uncapped, 5′-triphosphorylated RNA, characteristic of the 5′ ends of the genomic and antigenomic RNAs of some RNA viruses, as well as those of some viral transcripts (28); for reviews on the mechanism of action of IFIT1 and the IFIT family of proteins, see references 21, 26, 27, and 29. However, recent evidence suggests that there are differences in the mechanisms of action of the murine and human paralog IFIT1 proteins. While murine IFIT1 (IFIT1B) inhibits the translation of mRNAs that lack cap 1, it has been proposed that human IFIT1 recognizes some other, as-yet-undefined structure near the cap or possibly that 5′ mRNA sequences may help define the specificity of inhibition by human IFIT1 (30). The RNA-capping activity of viral RNA polymerases often include 2′-O-methyltransferases (2′-O-MTases), which modify cap 1 and thus can avoid inhibition by IFIT1(B), as evidenced by the sensitivity of virus mutants that lack 2′-O-MTase activity (for reviews, see references 21 and 26). Capping and methylation of viral RNAs are also important, as such modifications can prevent the activation of RIG-I, thereby reducing the amount of IFN produced by virally infected cells (for a review, see reference 31).

Here we have examined the ability of IFIT1 to inhibit the translation of a variety of paramyxovirus mRNAs and thus the replication of those viruses. We show that while all rubulaviruses tested were sensitive to IFIT1, all nonrubulavirus members of the Paramyxoviridae tested were insensitive. Lack of 2′ O-methylation of rubulavirus mRNAs was at least partially responsible for their inhibition by IFIT1. The possible biological consequences of differences in sensitivity of paramyxoviruses to IFIT1 are discussed.

## MATERIALS AND METHODS

### Cells, viruses, antibodies, and interferon.

A549 cells and derivatives were grown as monolayers in 25-cm^2^, 75-cm^2^, or 300-cm^2^ tissue culture flasks in Dulbecco's modified Eagle's medium supplemented with 10% fetal bovine serum at 37°C. When appropriate, cells were treated with human recombinant interferon (Intron A; Merck, Sharpe and Dohme) at 1,000 units/ml. Viruses used in these studies were PIV2 (strain Colindale), PIV3 (strains Washington and JS and recombinant ΔC and ΔD JS viruses [32]), PIV5 (formerly known as SV5; strains W3 [33] and CPI^+^ and CPI^−^ [34]), MuV (Enders [35]), respiratory syncytial virus (RSV) (36), Sendai virus (strain Cantell, free of defective interfering particles), and canine distemper virus (strain Mill Hill). Plaque assays were performed by standard methods in six-well dishes that included 0.1% Avicel (FMC Biopolymer) in the overlay medium. Plaques were visualized by immunostaining by using a pool of monoclonal antibodies or polyclonal antisera specific for the different viruses as described previously (37), together with alkaline phosphatase-conjugated secondary antibody by using SigmaFast BCIP/NBT as the substrate.

### Preparation of l-[^35^S]methionine-labeled total-cell extracts and SDS-PAGE.

Infected or uninfected cells that had or had not been pretreated with IFN for 12 h prior to infection were metabolically labeled for 1 h with l-[^35^S]methionine (500 Ci/mmol; MP Biomedical, USA) at 18 h postinfection (p.i.). After labeling, cells were lysed in disruption buffer, sonicated, and heated for 5 min at 100°C and then analyzed by gel electrophoresis (SDS-PAGE). The gels were fixed, stained, and dried, and resolved bands were visualized by phosphorimager analysis. When appropriate, the same amounts of cell equivalents were run on PAGE. Furthermore, the amount of protein in each sample was monitored by staining the polyacrylamide gels (PAGs) with Coomassie brilliant blue.

### Immunofluorescence.

Cells to be stained for immunofluorescence were grown on 10-mm-diameter coverslips (MIC3270; Scientific Laboratory Supplies, United Kingdom). Cells were stained with specific monoclonal antibodies (MAbs), as described in detail elsewhere (38). Briefly, monolayers were fixed with 5% formaldehyde–2% sucrose in phosphate-buffered saline (PBS) for 10 min at 20°C, permeabilized with 0.5% Nonidet P-40–10% sucrose in PBS for 5 min at 20°C, and washed three times in PBS containing 1% calf serum. PIV5- and PIV3-infected cells were detected by indirect immunofluorescence using a secondary goat anti-mouse Ig Texas Red-conjugated antibody (catalog number ab6787; Abcam). The primary antibodies were PIV5-NP-a and PIV5-Pe for PIV5 (39) and 4721, 2281, and 4812 for PIV3 (40). After staining for immunofluorescence, the monolayers of cells were examined with a Nikon Microphot-FXA immunofluorescence microscope.

### RNA selection and *in vitro* translation.

RNA for *in vitro* translations was isolated by sedimentation through CsCl gradients by a modified method described by Leppert et al. (41). Confluent monolayers of infected cells, grown in 300-cm^2^ flasks, were resuspended in ice-cold lysis buffer (150 mM NaCl, 50 mM Tris-HCl [pH 7.5], 0.6% NP-40, protease inhibitor cocktail [complete Mini EDTA-free, 1 tablet per 7 ml of buffer; Roche]) at 1 × 10^8^ to 2 × 10^8^ cells per ml and left on ice for 5 min prior to vortexing for 2 min. Nuclei were removed by centrifugation twice at 4,200 × *g* for 5 min at 4°C. The supernatant (cytoplasmic extract) was collected, made up to 6 mM EDTA, and layered onto 35% (wt/wt) CsCl in 25 mM Tris-HCl (pH 7.5)–2 mM EDTA followed by centrifugation at 175,000 × *g* at 12°C for 16 to 18 h. Naked RNA (including mRNA) forms a pellet at the bottom of the gradient, while viral genomic and antigenomic RNAs remain complexed with nucleoprotein and do not enter the 35% CsCl cushion. The supernatant was discarded, and the pellet was resuspended in RNase-free water and adjusted to 1 μg/μl. Selected RNA was translated *in vitro* with a rabbit reticulocyte lysate kit (L4960; Promega) in the presence of [^35^S]methionine-cysteine (NEG772, EasyTag Express Protein Labeling mix; PerkinElmer) using a modification to the manufacturer's instructions: methionine-cysteine-free medium (D0422; Sigma) was used to provide other amino acids (1 μl per 50 μl reaction mixture).

### Capping and methylation of mRNA.

Human RNA guanylyltransferase and 5′-phosphatase (RNGTT), RNA guanine-7 methyltransferase (RNMT), and cap methyltransferase 1 (CMTR1) were synthesized and purified according to the method of Gonatopoulos-Pournatzis et al. (42). As described in that study, the enzymes were all verified as being active by *in vitro* reactions followed by thin-layer chromatography. Capping and methylation reactions were carried out in 50 mM Tris-HCl (pH 8.0), 6 mM KCl, 1.25 mM MgCl_2_, 1 mM dithiothreitol (DTT) buffer as follows: 1 μl 10× buffer, 1 μl RNGTT (2.5 mg/ml), 1 μl RNMT (0.5 mg/ml), CMTR1 (0.28 mg/ml), 1 μl SAM (2 mM), 1 μl GTP (1 mM), 0.5 μl RNasin, 2 μl RNA (1 μg/μl). The reaction mixture was made up to 10 μl with H_2_O, including in experiments in which RNGTT, RNMT, or CMTR1 was omitted, and incubated at 37°C for 1 h.

### Cloning and purification of IFIT1.

IFIT1 was amplified with primer IFIT1F/IFIT1Xho from the plasmid pGAC-HA-IFIT1, restricted with NcoI and XhoI, and ligated with a modified pLOU3, in which maltose binding protein (MPB) was replaced with SUMO, while SalI in the mutliple cloning site (MCS) was replaced with XhoI. The primers were as follows: IFIT1F, CCGCCATGGCTACAAATGGTGATGATCATCAGG; IFIT1Xho, GCGCCTCGAGCTAAGGACCTTGTCTCACAGAGTT.

The fusion protein His-SUMO-(TEV)-IFIT1 was expressed in Escherichia coli strain Rosetta in 6 liters LB-ampicillin-chloramphenicol (LB/Amp/CM). Isopropyl-β-d-thiogalactopyranoside (IPTG; 0.2 mM) was added when an optical density (OD) of 0.8 was reached. The expression was carried out at 18°C overnight.

Purification was carried out with a routine protocol for His-tagged protein. The binding buffer contained 20 mM Tris-HCl (pH 8.0), 0.3 M NaCl, and 10 mM imidazole; the washing buffer contained 30 mM imidazole; and protein was eluted with 300 mM imidazole. To remove nonspecifically bound RNA, the columns were washed with 9 volumes of 0.2 M Na_2_HPO_4_–4 M NaCl (pH 7.5). After desalting into GF buffer (20 mM Tris-HCl [pH 8], 150 mM NaCl, 5% glycerol), the fusion was cleaved with tobacco etch virus (TEV) protease (1:100) at room temperature overnight. Gel filtration was carried out after passing through Ni beads again and addition of 3 mM DTT. The IFIT1 peak was collected and concentrated and had an *A*_260_/*A*_280_ of 0.7 to 0.8.

## RESULTS

### Paramyxoviruses interact in distinct ways with cells in a preexisting IFN-induced antiviral state.

**FIG 1:**
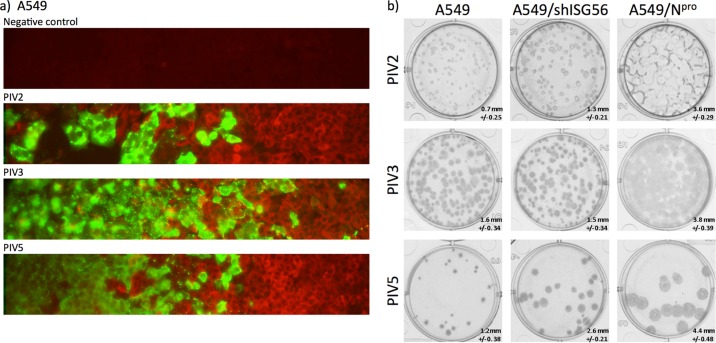
A549 cells produce and respond to IFN during development of PIV2, PIV3, and PIV5 plaques. (a) A549 cells grown on coverslips in 24-well microtiter plates were infected with PIV2, PIV3, or PIV5 at an MOI that resulted in 2 to 10 plaques per well. At 3 days p.i., the cells were fixed and stained with antibodies to the NP proteins of the respective viruses (green) and anti-MxA antibody, which is an ISG that is upregulated in response to IFN-α/β (red). The images show cross sections through plaques with the center of the plaque at the left-hand side of the image. (b) Relative plaque sizes of PIV2 (Colindale), PIV3 (Washington strain), and PIV5 (W3) on A549, A549/shIFIT1, and A549/NPro cells. Infected monolayers of cells in 6-well dishes were fixed at 4 days (PIV3) or 5 days (PIV2 and PIV5) p.i., and virus plaques were visualized by immunostaining the monolayers with antibodies to the respective NP and/or P proteins. The numbers at the bottom right in each panel give the average plaque sizes in millimeters with their standard deviations.

Despite the fact that paramyxoviruses encode IFN antagonists that inhibit IFN production and signaling, their ability to block the IFN response is not absolute. Thus, they form larger plaques on IFN-incompetent cells than IFN-competent cells (Fig. 1) (19), showing that during virus replication and spread some IFN is produced and slows the spread of the viruses (see Fig. 3). In the experiments shown in Fig. 1 and below, we used naive A549, A549/Npro, and A549/shIFIT1 cells; naive A549 cells can produce and respond to IFN in response to virus infection, and A549/Npro cells respond to exogenous IFN but cannot produce IFN as they constitutively express Npro from bovine viral diarrhea virus (BVDV), which targets IRF-3 for degradation (43). Furthermore, because IRF-3 is degraded in A549/Npro cells, they cannot upregulate expression of IFIT1 in an IRF-3-dependent, IFN-independent manner in direct response to virus infection (29). A549/shIFIT1 cells produce and respond to IFN, but expression of endogenous IFIT1 in response to IFN or viral infection is inhibited due to constitutive expression of small hairpin RNA (shRNA) to IFIT1 (23).

We previously showed that IFIT1 is the major cellular protein responsible for the IFN sensitivity of the rubulavirus PIV5 (23). To further investigate the role of IFIT1 and the ability of IFN to induce an antiviral state against other paramyxoviruses, we initially tested the ability of PIV2, PIV3, and PIV5 to form plaques in A549, A549/Npro, and A549/shIFIT1 cells. All three viruses induced IFN in A549 cells as the plaques developed, as observed by the induction of MxA in the uninfected cells surrounding the plaque (Fig. 1a). As previously observed (23), PIV5 formed bigger plaques on A549/shIFIT1 cells than on A549 cells, but the plaques were not as large as those on A549/Npro cells (Fig. 1b). While PIV2 also produced slightly larger plaques on A549/shIFIT1 than on A549 cells, the plaques on A549/Npro cells were obviously bigger (note that the center of mid- to large-sized PIV2 plaques has fallen out of monolayers). PIV3 produced similarly sized plaques on A549 and A549/shIFIT1 cells and slightly larger plaques on A549/Npro cells. These results also support our previous conclusion that in A549 (and Hep2) cells IFIT1 is the primary ISG effector to PIV5 (23) and that the rubulavirus PIV2 is also sensitive to IFIT1. However, knocking down IFIT1 did not have such a marked effect on PIV2 plaque size as it did for PIV5. This indicates that there are likely to be additional ISGs that play an important role in IFN-mediated inhibition of PIV2. In contrast, PIV3 (Washington strain) produced similarly sized plaques on A549 and A549/shIFIT1 cells and only slightly larger plaques on A549/Npro cells; this suggests that the IFN response is capable of slowing the spread of PIV3 to some degree (but not through the activity of IFIT1), but not as dramatically as it does for PIV2 or PIV5. However, experiments on the JS strain of PIV3 showed it to be more sensitive to the antiviral effects of IFN, but this was not because JS is sensitive to IFIT1 (data not shown).

**FIG 2:**
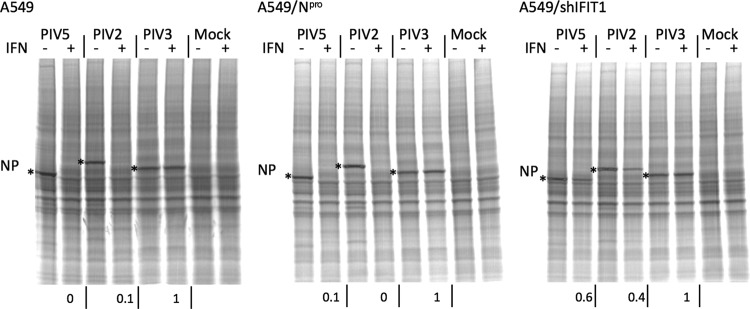
IFIT1 inhibits PIV5 (W3) and PIV2 (Colindale) protein synthesis but not that of PIV3 (Washington). A549, A549/NPro, and A549/shIFIT1 cells, grown in 25-cm^2^ flasks were, or were not, treated with IFN 8 h prior to infection at a high MOI with PIV2, PIV3 (Washington strain), or PIV5. At 18 h p.i., cells were metabolically labeled with [^35^S]methionine for 1 h. Total-cell extracts were separated by electrophoresis through a 4 to 12% PAG, and labeled proteins were visualized using a phosphorimager. The positions of the NP polypeptides are indicated by asterisks. The values at the bottom indicate the fraction of NP made in cells pretreated with IFN compared to untreated cells as estimated by densitometry scans.

We next compared the synthesis of viral proteins in cells infected with PIV2, PIV3, and PIV5 that had, or had not, been pretreated with IFN prior to infection with PIV2, PIV3, and PIV5. Cells were infected at a high multiplicity of infection (MOI; 10 to 20 PFU/cell), and the relative levels of NP synthesis were visualized by radioactively labeling the cells for 1 h with [^35^S]methionine at 18 h p.i. (Fig. 2). Pretreatment of A549 and A549/Npro cells with IFN in this assay reduced the expression of the NP of PIV2 and PIV5 to barely detectable levels. However, IFN pretreatment had no discernible effect on the expression of the NP protein of PIV3 or on the expression of host cell proteins. Strikingly, expression of NP of PIV2 and PIV5 was largely rescued in IFN-pretreated A549/shIFIT1 cells, demonstrating that IFIT1 plays a major role in the inhibition of PIV2 and PIV5 protein synthesis observed in A549 and A549/Npro cells pretreated with IFN. Figure 2 is an exemplar of many similar experiments that we have performed under different conditions (time course, MOI, etc.) and that show the same results, namely, that PIV2 and PIV5 are inhibited by IFIT1 while PIV3 is not.

**FIG 3:**
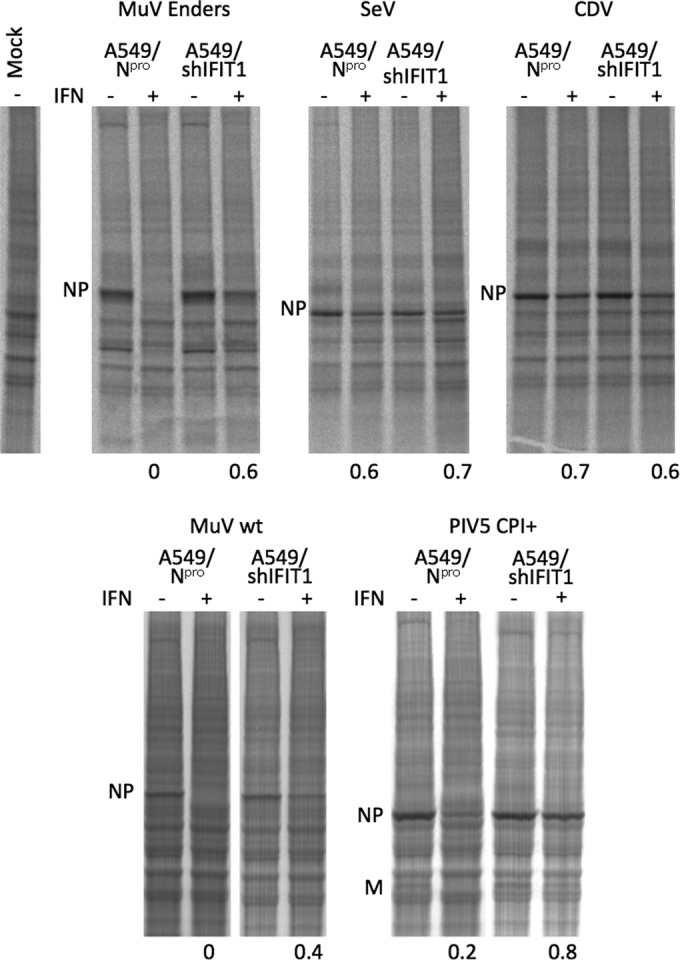
IFIT1 inhibits MuV (strain Enders and wild-type strain [Lo-1]) and PIV5 (strain CPI^+^) protein synthesis but not that of SeV or CDV. A549/N^Pro^ cells and A549/shIFIT1 cells, grown in 25-cm^2^ flasks, were or were not treated with IFN 8 h prior to infection at a high MOI with MuV Enders, MuV Lo-1 (wt), SeV, CDV, or PIV5 (CPI^+^). At 18 h p.i., cells were metabolically labeled with [^35^S]methionine for 1 h. Total-cell extracts were separated by electrophoresis through a 4 to 12% PAG, and labeled proteins were visualized using a phosphorimager. The values at the bottom indicate the fraction of NP made in cells pretreated with IFN to NP made in untreated cells.

Having demonstrated that PIV2 and PIV5 are sensitive to IFIT1 while PIV3 is resistant, we tested the sensitivity of other members of the Paramyxoviridae family, namely, mumps virus (MuV strain Enders), Sendai virus (SeV), and canine distemper virus (CDV). In a set of experiments similar to those illustrated in Fig. 2, A549/Npro and A549/shIFIT1 cells were or were not pretreated with IFN prior to infection with high MOI with these viruses. The relative levels of NP synthesis were visualized by radioactively labeling the cells for 1 h with [^35^S]methionine at 18 h p.i. (Fig. 3A). These experiments clearly demonstrated that, as was observed for PIV2 and PIV5, pretreating A549 cells with IFN inhibited MuV strain Enders protein synthesis but knocking down IFIT1 expression could largely restore MuV protein synthesis. In contrast, as was observed for PIV3, although pretreatment of A549 cells with IFN slightly reduced the expression of SeV and CDV protein synthesis, no increase in SeV and CDV protein synthesis was observed in A549/shIFIT1 compared to A549 cells pretreated with IFN. These results therefore show that MuV Enders is sensitive to IFIT1 but SeV and CDV are not, the weak inhibition of SeV and CDV protein synthesis observed in A549 and A549/shIFIT1 cells pretreated with IFN presumably being due to the action of other ISGs induced by IFN. While MuV is sensitive to IFIT1, it forms pinpoint plaques on A549/Npro cells only at 5 days p.i. (data not shown), strongly suggesting that there are host cell restrictions other than innate intracellular defense mechanisms on MuV replication in A549 cells (44).

Since in these experiments we used the attenuated Enders strains of MuV to test whether attenuation may be linked to sensitivity to IFIT1, we tested a wild-type (wt) isolate of MuV-London-1 (Lo-1) for its sensitivity. At the same time, we also tested the sensitivity of another strain of PIV5, termed CPI^+^ (Fig. 3B). MuV-Lo was as sensitive as MuV Enders, demonstrating that attenuation was not linked to differences in their relative sensitivity to IFIT1. Similarly, PIV5 CPI^+^ was also sensitive to inhibition by IFIT1.

### The IFIT1 sensitivity of PIV5 is not rescued by coinfection with an IFIT1-resistant virus.

**FIG 4:**
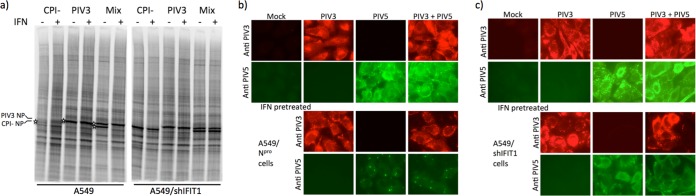
PIV3 (Washington) does not inhibit the antiviral activity of IFIT1. A549 and A549/shIFIT1 cells, grown in either 25-cm^2^ flasks (a) or on coverslips (b and c), were or were not treated with IFN 8 h prior to infection at a high MOI with PIV3, PIV5 (strain CPI^−^), or a mixture of the two viruses. (a) At 18 h p.i., the cells were metabolically labeled with [^35^S]methionine for 1 h. Total cell extracts were separated by electrophoresis through a 4 to 12% PAG, and labeled proteins were visualized using a phosphorimager. The positions of the NP protein are indicated by asterisks. (b and c) At 18 h p.i., cells grown on coverslips were fixed and immunostained with antibodies specific for the NP and/or P proteins of the respective viruses.

From these results, it was clear that replication of the nonrubulaviruses PIV3, SeV, and CDV is not inhibited by IFIT1. To investigate whether PIV5 replication could be rescued by coinfections with an IFIT1-resistant virus, mixed infections between PIV3 and PIV5 were undertaken. To avoid any possible synergistic effects between PIV3 and PIV5 in dismantling an IFN-induced antiviral state, the CPI^−^ strain of PIV5 was used in these experiments because, due to mutations in its V protein, it does not block IFN signaling (45). A549 or A549/shIFIT1 cells were or were not pretreated with IFN for 8 h prior to high-MOI (10 to 20 PFU/cell) infection with PIV5, PIV3, or a mixture of the two viruses (Fig. 4a). The expression of the NP protein of PIV3 was resistant to IFN in both A549 and A549/shIFIT1 cells when they were infected with PIV3 alone and when coinfected with PIV5. In contrast, while the expression of PIV5 NP was resistant to IFN in A549/shIFIT1 cells, its expression was inhibited in A549 cells, even when the cells were coinfected with PIV3. Immunofluorescence was undertaken to ensure that in these experiments there was no exclusion of one virus by the other (Fig. 4b and c). These results confirmed that coinfection of PIV3 with PIV5 does not rescue the sensitivity of PIV5 to IFIT1 and strongly suggest that PIV3 does not specifically inhibit the antiviral activity of IFIT1 and that the inhibition of PIV5 NP expression is regulated by *cis*-acting elements.

### Differential inhibition of translation of mRNAs of different paramyxoviruses by purified IFIT1.

**FIG 5:**
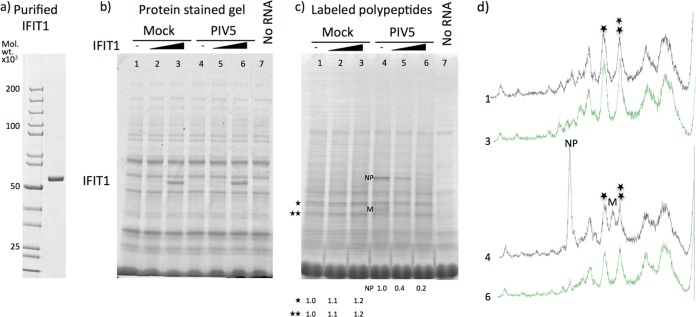
Purified IFIT1 directly inhibits *in vitro* translation of PIV5 mRNA. (a) Coomassie brilliant blue-stained PAG of purified IFIT1; molecular weight markers are shown in the left-hand lane. (b and c) RNA isolated from mock- or PIV5-infected cells was *in vitro* translated in the presence of [^35^S]methionine for 90 min in the presence or absence of increasing concentrations of purified IFIT1 (0.1 and 1.0 μg per reaction mixture). Polypeptides were separated by electrophoresis through a 4 to 12% PAG. The total protein contents present in the *in vitro* translation mixes were visualized by staining the gel with Coomassie brilliant blue (b), and the radioactively labeled proteins were visualized using a phosphorimager (c). (d) Densitometry traces of lanes 1, 3, 4, and 6 of panel c. The positions of PIV5 NP and M proteins are indicated; asterisks mark two prominent host cell polypeptides. The values at the bottom of the gel indicate the fraction of either the host cell proteins or NP proteins made in the *in vitro* translation mixes in the presence of purified IFIT1 compared to those made in the absence of IFIT1.

The data above show that the IFN sensitivity of rubulaviruses is at least in part due to the actions of IFIT1. Since this cellular protein has been shown to inhibit translation in a template-specific manner, we developed an *in vitro* translation system to study the ability of human IFIT1 to selectively inhibit the translation of rubulavirus mRNAs. The gene encoding human IFIT1 was cloned as a SUMO fusion protein expressed in Escherichia coli, and the recombinant protein was purified (Fig. 5a). To determine whether the recombinant IFIT1 was able to selectively inhibit PIV5 mRNAs, *in vitro* translation of mRNA isolated from mock- and PIV5-infected cells was carried out in the presence and absence of different concentrations of IFIT1 (Fig. 5b, c, and d). In the absence of IFIT1, expression of the NP protein (and to a lesser extent the M protein) of PIV5 was clearly visualized in the background of *in vitro*-translated cellular proteins (Fig. 5c and d). Increasing concentrations of IFIT1 had no obvious effect on the efficiency of translation of host cell proteins, but in striking contrast, purified IFIT1 selectively inhibited the translation of the NP and M proteins of PIV5 in a concentration-dependent manner.

**FIG 6:**
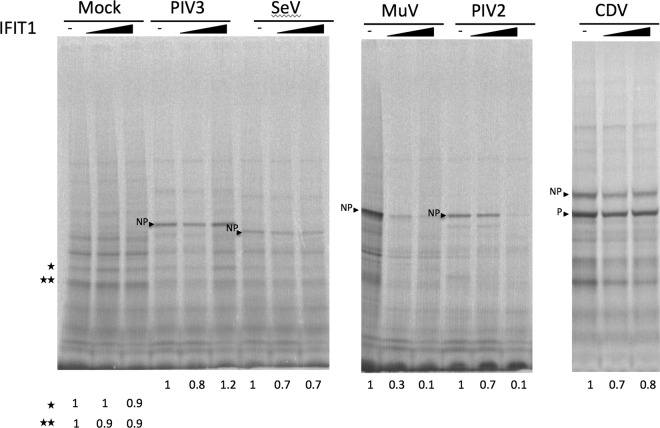
Purified IFIT1 inhibits the *in vitro* translation of NP mRNA isolated from PIV2- and MuV-infected cells but not mRNA from mock-infected cells or NP mRNA isolated from PIV3 (Washington strain)-, SeV-, or CDV-infected cells. RNA isolated from mock-infected or infected cells was *in vitro* translated in the presence of [^35^S]methionine for 90 min in the presence or absence of increasing concentrations of purified IFIT1 (0.1 and 1.0 μg per reaction mixture). Polypeptides were separated by electrophoresis through a 4 to 12% PAG, and labeled proteins were visualized using a phosphorimager. The positions of the NP proteins are indicated. The numbers at the bottom of the gel indicate the fraction of either the host cell proteins or NP proteins made in the *in vitro* translation mixes in the presence of purified IFIT1 compared to those made in the absence of IFIT1.

Having established that the sensitivity of *in vitro* translation of PIV5 mRNA to inhibition by purified IFIT1 correlated with the biological sensitivity of PIV5 to IFIT1, we next tested the ability of IFIT1 to inhibit the translation of mRNA isolated from cells infected with other paramyxoviruses (Fig. 6). These results clearly demonstrated that translation of (NP) mRNAs from PIV2- and from MuV-infected cells was inhibited by IFIT1. In contrast, there was no obvious reduction in the amount of PIV3 NP synthesized when increasing amounts of IFIT1 was added to the *in vitro* translation reaction mixtures. Although there was a slight apparent reduction in the amount of SeV and CDV NP synthesis in the samples in which IFIT1 was added, there was no increase in the inhibition observed by increasing the amount of IFIT1 added to the *in vitro* translation reaction mixtures, strongly suggesting that the translations of SeV and CDV mRNAs are also resistant to inhibition by IFIT1.

### Lack of 2′-O methylation of the cap structure of MuV and PIV5 mRNAs is partially responsible for their sensitivity to inhibition by IFIT1.

**FIG 7:**
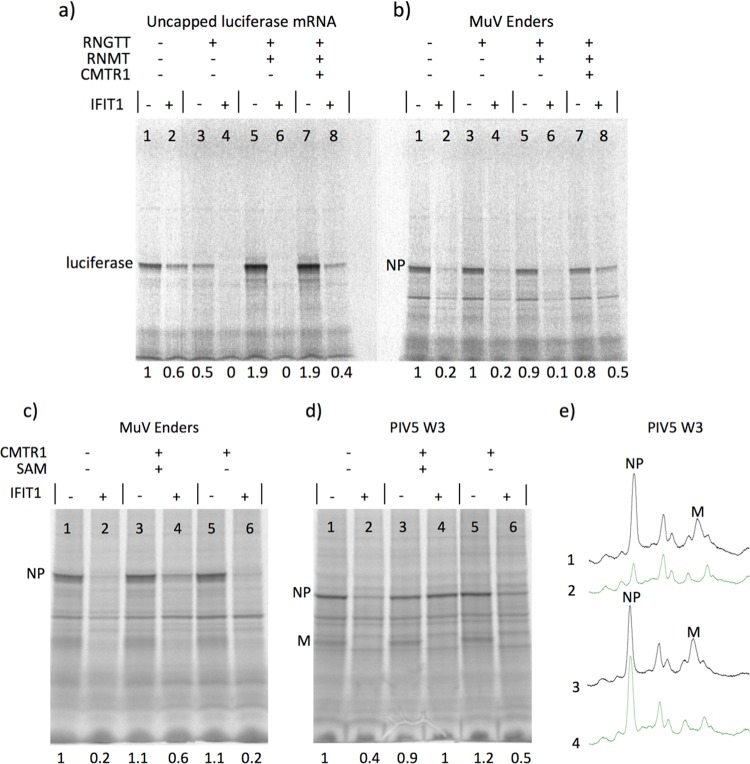
Lack of 2′-O methylation of the cap 1 structure of MuV and PIV5 mRNAs is at least partially responsible for their sensitivity to inhibition by IFIT1. (a) Uncapped 5′-ppp mRNA encoding luciferase synthesized by T7 polymerase (provided as a control in the Promega *in vitro* translation kit) was translated *in vitro* in a rabbit reticulocyte lysate in the absence or presence of purified IFIT1 (lanes 1 and 2). RNGTT was used to add a 5′ guanine cap (lanes 3 and 4); then, RNMT was used to methylate the cap at the N7 position (lanes 5 and 6), generating cap 0, and CMTR1 was used to methylate the adjacent ribose on the 2′ OH position, generating cap 1. The modified mRNAs were then *in vitro* translated in the absence (lanes 3, 5, and 7) or presence (lanes 4, 6, and 8) of IFIT1. (b) mRNA isolated from MuV-infected cells was treated in parallel under the same conditions as those described for panel a. (c and d) mRNA isolated from either MuV (Enders)- or PIV5 (W3)-infected cells was *in vitro* translated prior to (lanes 1 and 2) or following (lanes 3 and 4) modification by CMTR1 in the presence or absence (lanes 5 and 6) of SAM. The mRNA was also translated in the absence (lanes 1, 3, and 5) or presence (lanes 2, 4 and 6) of purified IFIT1. (e) Densitometry traces of lanes 1, 2, 3, and 4 of panel d. The numbers at the bottom of the gel indicate the fraction of either the host cell proteins or NP proteins made in the *in vitro* translation mixes in the presence of purified IFIT1 compared to those made in the absence of IFIT1.

Previous studies have shown that the absence of cap 1 on mRNAs renders them sensitive to inhibition to IFIT1. To investigate whether this was the case for rubulavirus mRNAs, we developed an *in vitro* assay in which purified human mRNA-modifying enzymes were used to progressively cap and add different methyl groups to the 5′ ends of mRNAs. Purified human RNA guanylyltransferase and 5′-phosphatase (RNGTT), RNA guanine-7 methyltransferase (RNMT), and cap methyltransferase 1 (CMTR1) were used in these assays. RNGTT adds a 5′ guanosine to RNAs with 5′-ppp, while RNMT adds a methyl group to the ^7^G of the guanine ring, generating (m^7^G) cap 0. CMTR1 adds a methyl group to the 2′ OH position of the adjacent ribose, generating cap 1. To demonstrate the functionality of this system, we first tested the *in vitro* translation of luciferase mRNA with a 5′-triphosphate group. This RNA was efficiently translated in a cap-independent manner and was only weakly inhibited by IFIT1 (Fig. 7a, compare lanes 1 and 2). When the luciferase mRNA was capped with the addition of 5′-guanosine by RNGTT (generating Gppp-mRNA), there was a slight decrease in the amount of luciferase made (Fig. 7a, compare lanes 1 and 3). This may have been due to RNGTT destabilizing or blocking the translation of Gppp-mRNAs in the absence of ^7^N methylation. However, strikingly, translation of this mRNA was completely inhibited by IFIT1 (Fig. 7a, lane 4) despite this cap structure lacking N-7 methylation. As expected, the addition of a methyl group to the N-7 position of the guanine ring, generating m^7^Gpppm^2^N, by RNMT increased the efficiency of translation, but m^7^Gppp-luciferase remained completely sensitive to inhibition by IFIT1 (Fig. 7a, lanes 5 and 6). Addition of a methyl group to the 2′ OH group of the adjacent ribose, generating cap 1, by CMTR1 did not affect the efficiency by which the mRNA was translated, but it did clearly reduce the sensitivity of the mRNA to inhibition by IFIT1 (Fig. 7A, compare lanes 7 and 8). However, it should be noted that in these experiments, for reasons that are unclear, we were unable to completely restore full translation of the luciferase mRNA in the presence of IFIT1 by increasing the amount of CMTR1 or the length of incubation of the mRNA with the enzyme (data not shown).

To investigate how similar modifications to the cap of rubulavirus mRNAs influenced their inhibition by IFIT1, we initially used MuV mRNA in a parallel set of experiments. These results showed that treatment of the MuV mRNA with RNGTT and RNMT did not increase the efficiency of *in vitro* translation of MuV NP mRNA or its sensitivity to inhibition by IFIT1 (Fig. 7b, lanes 1 to 6), consistent with the viral polymerase adding m^7^Gppp-cap at (cap 0) to the 5′ end of viral mRNAs. However, surprisingly, since rubulavirus polymerases have conserved 2′-O MTase domains, addition of a methyl group to the 2′OH group of the adjacent ribose (cap 1) by CMTR1 clearly reduced the sensitivity of the NP mRNA to inhibition by IFIT1 (Fig. 7b, lanes 7 and 8). As expected, the IFIT1 sensitivity was dependent on the addition of S-adenosyl methionine (SAM) to the reaction mixture (Fig. 7c). Similarly, following 2′O methylation of PIV5 mRNA, *in vitro* translation of PIV5 NP became completely resistant to inhibition by IFIT1 (Fig. 7d). Strikingly, in contrast to NP, the translation of PIV5 M mRNA remained completely sensitive to inhibition by IFIT1 even after 2′O methylation of PIV5 mRNA by CMTR1 (Fig. 7d and e); the basis for this is currently unknown, but we are investigating it further.

## DISCUSSION

Over the past decade or so, it has become clear that the ways in which paramyxoviruses circumvent innate immune responses, including the IFN response, and differences in the multifunctional nature of their IFN antagonists are likely to influence the types of disease they cause. For example, the viral IFN antagonists within the rubulavirus genus, namely, the V proteins, as well as interacting with common targets such as MDA 5 and LGP2 also have unique properties. The V protein of PIV5 targets STAT1 for degradation, PIV2 targets STAT2, and MuV targets both STAT1 and STAT3. Within the Respirovirus and Morbillivirus genera, it is a combination of the V and C proteins that counteract innate responses by different molecular mechanisms, and strikingly, although PIV3 encodes a C protein, it does not encode a functional V protein. Despite encoding of these powerful IFN antagonists, IFN is produced during virus spread both in tissue culture cells and *in vivo*, and thus undoubtedly paramyxoviruses will, during the course of an infection, infect cells in a preexisting IFN-induced antiviral state. Here we show that different paramyxoviruses interact in distinct ways with cells in a preexisting IFN-induced antiviral state, and we suggest that this may influence the types of diseases caused. Strikingly, in contrast to the sensitivity of rubulaviruses to IFIT1, the other paramyxoviruses that we tested were resistant, strongly suggesting that this might be a distinguishing feature of rubulaviruses, although before this can be firmly concluded the sensitivity of more species of paramyxoviruses to IFIT1 needs to be tested. Even within the Rubulavirus genus, it appears that there may be differences in how members interact with cells in an IFN-induced antiviral state. In A549 cells, IFIT1 primarily is responsible for the IFN-induced antiviral state induced to counter PIV5. However, although PIV2 is sensitive to IFIT1, there appear to be other ISGs that have strong anti-PIV2 activity. This conclusion comes from the observation that while there is a slight increase in the size of PIV2 plaques on A549/shIFIT1 cells compared to A549 cells, it is not as obvious as that observed for PIV5. Furthermore, while plaques for PIV5 were smaller on A549/shIFIT1 cells than on A549/Npro cells, this difference was not as marked as that observed for PIV2. MuV strain Enders is also sensitive to IFIT1, but there are clearly other major constraints on the growth of MuV Enders in human cells, as the virus grows extremely poorly in IFN-incompetent human cells but replicates to high titers in Vero cells (44).

It is striking that only rubulaviruses are sensitive to the antiviral activity of human IFIT1. Our data indicate that the inhibition of rubulavirus mRNAs was produced by IFIT1 in a *cis*-linked manner, implying that the restriction is associated with some feature of the mRNA sequence or structure. Since IFIT1 can selectively inhibit the translation of mRNAs that are incorrectly capped or not methylated at the 2′ OH group of the first ribose, i.e., cap 1 (24, 46, 47), it was likely that rubulaviruses have a structural motif in their cap, not present or hidden in the mRNA of other paramyxoviruses, that is recognized by IFIT1. To investigate this further, we used purified human enzymes to modify the cap of mRNAs. As a control for the activity of the enzymes, we used an uncapped 5′-ppp mRNA that encodes luciferase. The 5′-ppp luciferase mRNA translated in a cap-independent manner *in vitro* using rabbit reticulocyte lysate, and this translation was only weakly inhibited by purified IFIT1. While addition of a 5′ guanosine nucleoside cap slightly decreased the amount of luciferase synthesized, probably because the enzyme RNGTT destabilizes the mRNA, addition of the (unmethylated) guanosine nucleoside to the 5′ end of the mRNA significantly increased the sensitivity of the mRNA to inhibition by IFIT1. Furthermore, although methylation of the guanine ring at position N7 (m7GpppNp-RNA) by RNMT increased the efficiency of translation of luciferase mRNA, it did not appear to affect the sensitivity of inhibition by IFIT1. These results are therefore consistent with the observation that human IFIT1 binds with low affinity to 5′-ppp RNA but more avidly to cap 0 RNA lacking 2′ O methylation. Methylation at position N7 of the guanine ring has also been reported to increase the affinity of binding of IFIT1 (24); however, the observation here that Gppp-luciferase is inhibited as efficiently as m7Gppp-luciferase suggests that the methyl group does not play a central role in the inhibition of mRNAs by IFIT1. In contrast, 2′ O-methylation of the first ribose by CMTR1 to generate cap 1 partially prevented IFIT1 from inhibiting the translation of the cap 0-modified mRNA. However, even by increasing the amount of CMTR1 and the incubation time, we were unable to completely restore full translational activity of the luciferase mRNA. The reasons for this are unclear, but it suggests that other structural features, for example, methylation of the penultimate ribose to generate cap 2 or sequences at the 5′ end of mRNAs, may also influence inhibition by IFIT1, as has been suggested by Daugherty et al. (30).

mRNAs isolated from PIV3-, SeV-, and CDV-infected cells were not inhibited by IFIT1, and neither was the replication of these viruses (Fig. 2 and 3). In contrast, 2′ O-methylation of the terminal ribose by CMTR1 of MuV mRNAs partially alleviated inhibition of the NP mRNA by IFIT1. With regards to PIV5, our previous studies suggested that PIV5 mRNAs were 2′ O-methylated (23). Furthermore, we never observed complete IFIT1 inhibition of PIV5 NP synthesis *in vitro*, suggesting that at least a proportion of the PIV5 NP mRNA was correctly capped. However, the fact that treating PIV5 mRNAs with CMTR1 rescued NP synthesis in the presence of IFIT1 suggests that a significant proportion of PIV5 mRNAs was also not fully methylated. It is also of potential significance that the M mRNA of PIV5 appears to be more sensitive than NP mRNA to inhibition by IFIT1, and furthermore, translation inhibition of PIV5 M mRNA was not rescued by treatment with CMTR1. The differences in the relative sensitivity of the NP and M mRNAs clearly warrant further investigation but may be due to the fact that the viral methyltransferase differentially methylates the viral mRNAs (as has been shown for vesicular stomatitis virus [VSV] [48]), that CMTR1 does not recognize the untranslated region (UTR) of the PIV5 M mRNA, or that inhibition by IFIT1 is influenced by additional structural features present on PIV5 M mRNA but not NP mRNA. Regarding the latter point, it is of note that the first three nucleotides of the UTRs of NP and M differ. Furthermore, the 4 or 5 nucleotides downstream of cap 0 are thought to be bound by IFIT1 and may thus modulate IFIT1-RNA interactions (49), and some secondary RNA structures, e.g., those found at the 5′ end of some alphaviruses, can prevent IFIT1 binding to RNA independent of the cap methylation status (50).

Most viruses successfully avoid inhibition by IFIT1 by encoding their own 2′-O MTase, by cap snatching appropriately capped and 2′-O methylated structures from cellular mRNAs, or by having cap-independent translation with the covalently linked viral protein VPg or a 5′ RNA secondary structure that blocks the activity of IFIT1 (reviewed in reference 26). Indeed, work on virus restriction by IFIT1 has involved primarily the investigation of viruses in which the 2′-O-MTases have been mutated such that their mRNAs do not have a cap 1 structure (25, 51–54). Nevertheless, our results show that the viral polymerase of rubulaviruses, unlike other paramyxoviruses, does not fully protect the viral mRNAs from inhibition by human IFIT1. In this regard, it is of interest that although rubulaviruses have the conserved methyltransferase domain in their polymerase, they all have an alanine instead of the first glycine in a GxGxG motif present in the methyltransferase domain of other paramyxoviruses and mononegavirales, which has been shown to affect the efficiency of cap methylation (55).

Most viruses, including other mononegavirales (56), appear to be naturally resistant to inhibition by IFIT1. It is therefore intriguing that rubulaviruses have not evolved mechanisms to ensure that their mRNAs are correctly capped and methylated or have the appropriate UTRs to be resistant to IFIT1. It is tempting to speculate that there is some unknown biological advantage to being sensitive to IFIT1. For example, it may help some rubulaviruses (and perhaps hepatitis C virus [57], which is also sensitive to IFIT1) to establish prolonged or persistent infections. Thus, following infection of cells in an IFN-induced antiviral state, IFIT1 restricts PIV5 replication. Under such conditions, virus genomes are located in cytoplasmic foci, where, as we have previously suggested, they may remain hidden from intracellular and adaptive immune responses. Furthermore, if viral mRNA is produced in cells in an IFN-induced antiviral state, then viral protein synthesis will largely be inhibited by IFIT1, thus reducing the amount of protein that may be processed and presented to cytotoxic T lymphocytes (CTLs). Eventually, in such cells, however, enough of the virus IFN antagonist, the V protein, will be produced or brought in by infecting virus particles to target STAT1 for proteasome-mediated degradation, and the cells will no longer be able to maintain their antiviral state, thus facilitating virus replication (58). Whether such a scenario occurs *in vivo*, these and other considerations emphasize that to fully understand the molecular pathogenesis of viruses, it will be necessary to understand the subtleties of how viruses interact with the IFN system and other host cell defense mechanisms.
